# Supplementary material for: Establishment and Verification of a Novel Gene Signature Connecting Hypoxia and Lactylation for Predicting Prognosis and Immunotherapy of Pancreatic Ductal Adenocarcinoma Patients by Integrating Multi-Machine Learning and Single-Cell Analysis
Source: Int J Mol Sci. 2024 Oct 17;25(20):11143. doi: 10.3390/ijms252011143 (PMC11508839; doi:10.3390/ijms252011143)
Supplement: Supplementary file 1 [file ijms-25-11143-s001.zip › ijms-3199673-supplementary.pdf]

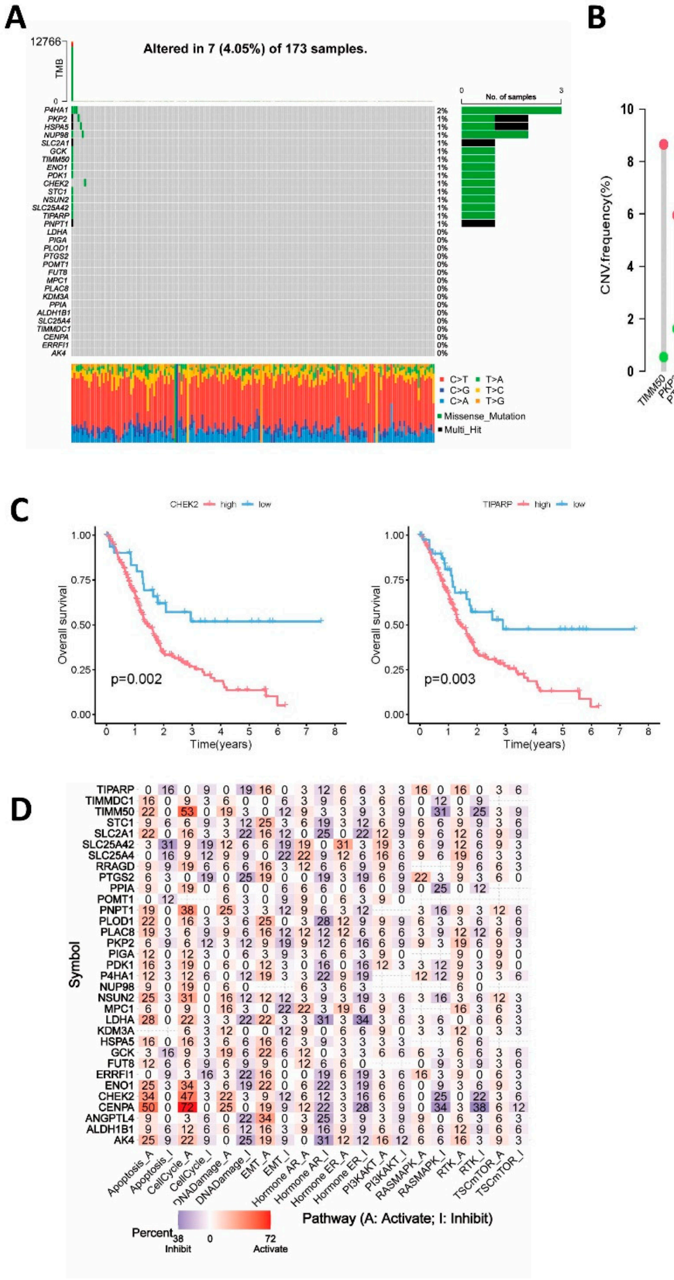

Supplementary Figure S1| (A,B) Mutation frequency of these prognostic genes in PDAC patients from the TCGA cohort. (C) Kaplan-Meier survival curve of prognostic genes. (D) Proportion of cancer cases where specific gene mRNA expression potentially impacts pathway activity.

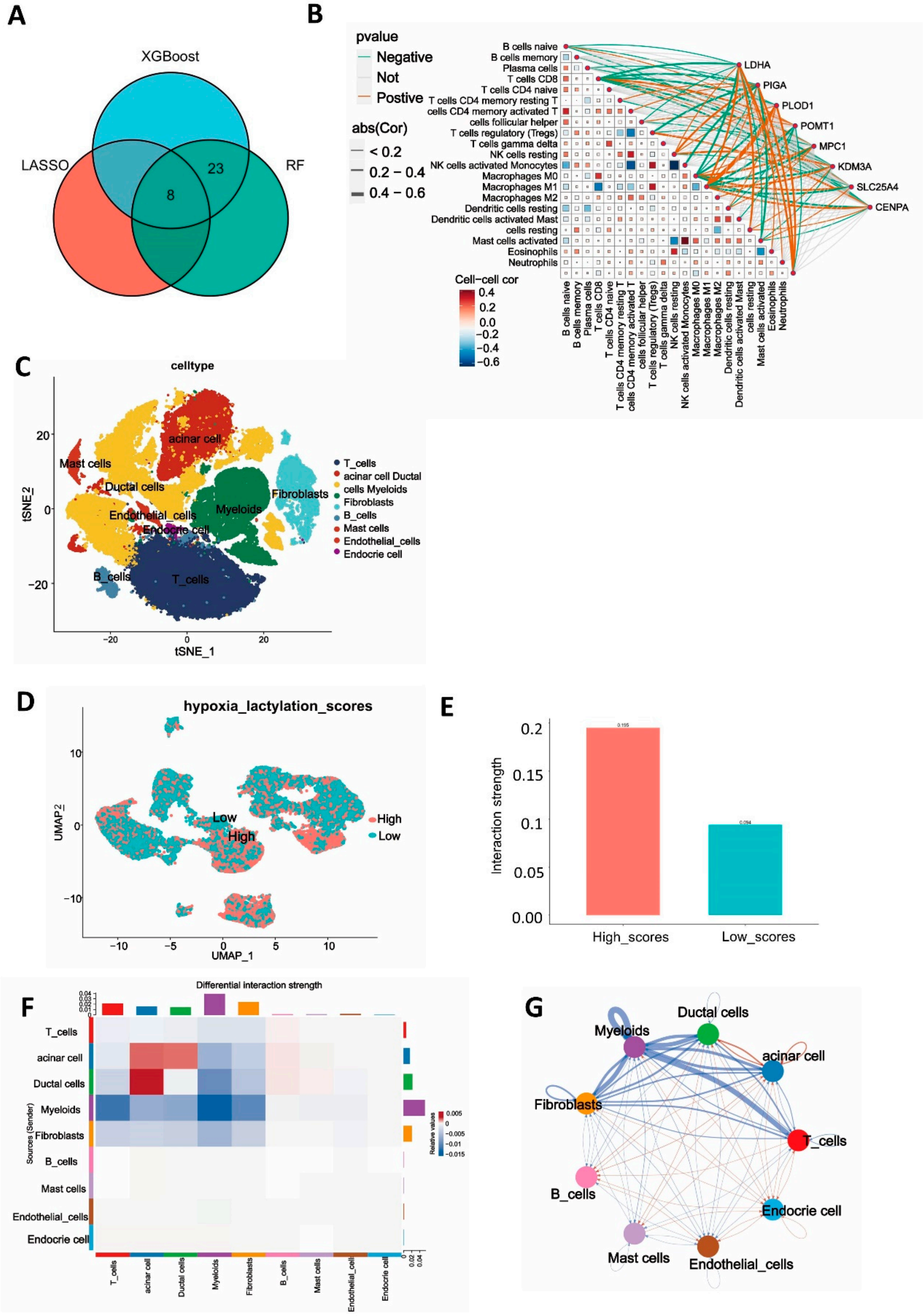

Supplementary Figure S2|(A) Venn diagram illustrates the overlap of hub genes identified by three supervised machine learning approaches. (B) Correlation between signature genes and infiltrating immune cells analyzed by the CIBERSORT algorithm. (C) t-SNE plot showing nine cell subtypes identified from PDAC scRNA-seq data. (D) UMAP-1 plot showing distribution of high and low hypoxia_lactylation_scores in primary PDAC

scRNA samples. (E) Bar plot showing interaction strength of high and low-scoring groups. (F,G)Heatmap and circular plot visualizing differences in cell-cell communication networks between high and low-scoring groups.
